# Termination of Pregnancy in Curaçao: Need for Improvement of Sexual and Reproductive Healthcare

**DOI:** 10.5539/gjhs.v4n3p30

**Published:** 2012-05-01

**Authors:** Adriana A. Boersma, Jantina F. Alberts, Jeanne de Bruijn, Betty Meyboom-de Jong, Gunilla Kleiverda

**Affiliations:** 1 General Practice, Breedestraat (O) 33-35, Willemstad, Curaçao; 2 Medical Sociologist, Foundation Handicapped and Rehabilitation Care, Willemstad, Curaçao; 3 Department of Social and Behavioral Sciences, University of the Netherlands Antilles, Willemstad, Curaçao; 4 Department of General Practice, University Medical Center Groningen, The Netherlands; 5 Department of Obstetrics and Gynecology, Flevo Hospital, Almere, The Netherlands

**Keywords:** termination of pregnancy, abortion, unintended pregnancy, contraception, curaçao, caribbean region

## Abstract

**Background:**

In Curaçao Termination of Pregnancy (TOP) is still forbidden by law, although a policy of tolerance has been stipulated since 1999. This paper is about the prevalence of TOP and about its health complications. These data on TOP are officially unknown but are suspected to be rather high.

**Methods:**

One year registration of illegal performed termination of pregnancy cases by all general physicians (GPs) practicing TOP in Curaçao. The registration included patient characteristics according to the model of the National Abortion Registration in The Netherlands, adjusted to the local Curaçao situation. Socio demographic characteristics, number of previous pregnancies and TOPs, pregnancy duration, contraception methods and reason for failure were registered. The comparative part of the research compares TOP rates of Curaçao with those of Antillean women in the Netherlands. The gynaecologists in the referral hospital registered complications requiring hospital admission after TOP.

**Results:**

All GPs performing TOP participated and the majority registered extensively. The total number of registered TOP was 1126. 666 of the 1126 were registered using the local adjusted Abortion Registration Model. With 30.000 women aged between 15 and 45 living in Curaçao, the TOP rate was at least 38 (per 1000 in that age category), comparable to rates for Antillean women in the Netherlands. Mean age was 26.9 years. Nearly half (47%) had one or more TOPs before; the majority (53%) was less than 7 weeks pregnant and two third (67%) had one or more children. Two third of the women did not use contraception (63%). For those using contraception, main reason for failure was inconsistent use (50%). There were 14 hospital admissions due to complications of TOP.

**Conclusion:**

The number of TOP is high in Curaçao and comparable to (first generation) Antillean women living abroad in the Netherlands. Most unintended pregnancies originated from no or inconsistent use of reliable contraception. Improvement of sex education is necessary in order to bring down the number of TOP, as well as realizing accessible and affordable contraception, including sterilization. The number of complications around TOPs was equal to other countries where TOP is illegal.

## 1. Introduction

Curaçao, a Caribbean island, part of the Kingdom of the Netherlands, is situated near Venezuela, on the edge of Latin America. The island is predominantly Roman Catholic and has an autonomous status and independent legislation, in which termination of pregnancy (TOP) is completely prohibited by law. Remarkable is the sharp contrast in two types framing of TOP in this small island society. On the *one* hand TOP is framed as a strong moral issue, as *a taboo topic* and forbidden by religion (Burns, 2005). In moral sense there is no space, termination of pregnancy is wrong. The island culture is conflict avoiding on all sensitive subjects or controversial materials such as termination of pregnancy. Such issues are de-emphasized or omitted. Open discussions are widely avoided, in the media, on the schools and in public forums. Sensitive topics receive little attention in schools because in the culture of schooling, and the culture of society, controversial topics and issues are taboo, a Polynesian word that means a general ban on the specific object, which should not be touched, in other not to bring society out of balance (Evans, Avery, & Pederson, 1990). This is in sharp contrast with the *second* type of framing of TOP as a *common* *medical practice*, which is expected to be something totally different from the moral framing. This second medical type of framing is as much alive in Curaçao as is the moral type of framing. In the medical framing, TOP must be done safely, quickly and without much discussion with whom it concerns. It is a broadly and easy used medical practice on the island. TOP is used without much hesitation, neither from most GPs nor from the unintended pregnant women. This medical framing of TOP fits easily into the modern globalizing culture of Curaçao in terms of thinking about women’s rights and reproductive rights. This extreme contradiction in the culture makes it just possible for general practitioners (GPs) to perform TOP and for women to obtain TOP as long as the discussion about TOP is avoided, otherwise it will be vetoed (Pheterson & Azize, 2005). TOP on request of a woman is performed by a small number of general practitioners. The Health Inspectorate and the Public Prosecutor’s office are aware of it. The Public Prosecutor stipulated a tolerance policy in 1999 and the Inspectorate supervises correct compliance. In this “institutionalized tolerance” policy the health personnel involved in TOP care is protected from prosecution. Since 1999 GPs carrying out TOP have no longer been prosecuted. Women having TOP are not protected, but none of them has been prosecuted because of TOP since then. Still, due to the highly restrictive law TOP is a criminal act. A change in law still seems far away because of the strong influence of the Roman Catholic Church and, for most policy makers, TOP is still a highly sensitive subject. Like in other countries, in times of highly political debates on abortion and contraceptives, it is almost impossible to liberalize the laws (Burns, 2005).

The medical practice in Curaçao first trimester illegal TOP (instrumental as well as medical) on request is carried out by 11 of the 102 GPs. Instrumental TOP means aspiration of the uterine contents. Medical TOP is performed with mifepristone (a progesterone receptor blocker) or methotrexate (a folic acid antagonist, interfering DNA synthesis and cell proliferation) and misoprostol (a prostaglandine E1 analogue) (Bartz & Goldberg, 2009). These GPs practice this in addition to their regular work. Women’s access to health care by GPs is universal for those with a health insurance (approximately 90% of the population). Because TOP is not included in health insurance, women have to pay the GP for the procedure. There is a lack of information by Public Health Services about TOP and not all (para-) medical practitioners will refer to a TOP providing GP because of their moral objections against TOP. This causes sometimes unnecessary postponement of the desired procedure. The eight gynaecologists practicing in the main hospital in the capital of Curaçao perform TOP in case of fetal or maternal reasons after approval of the ethical board of the hospital: curettage is performed till 12 weeks and misoprostol is used for second trimester TOP. Maternal reasons for TOP include women becoming pregnant but suffering severe diseases like heart or kidney diseases, sickle cell anemia, diabetes or severe hypertension to avoid serious health complications. Fetal defects having a great likelihood of causing death within a year after birth are fetal reasons for TOP. Also TOPs for these reasons are not legal but part of the Public Prosecutors’ tolerance policy. The gynaecologists treat as well complications after TOP performed by GPs or by women themselves. There are no TOP providing gynaecologists practicing outside the main hospital, neither are there gynaecologists treating complications of TOP in other hospitals.

In case of TOP outside the medical setting, the use of misoprostol is also prevalent in Curaçao. Misoprostol is a Prostaglandine E1 analogue with uterotonic and cervical-ripening effects. It promotes expulsion of the fetus. As in other South and Central American countries where TOP also mostly is prohibited, women make use of misoprostol which is easily accessible (Alan Guttmacher Institute, 2003). The non-legal situation of TOP, the moralistic framing that makes the issue a taboo for discussion, the shame, the lack of accessible and affordable reproductive health services contribute to this outside medical setting TOP practice with increased risk of complications.

Many first and second generation women from Curaçao live in the Netherlands, a country with a low TOP rate (8.7 per 1000 women in the reproductive age). First generation women from Curaçao are born in Curaçao and emigrated to the Netherlands. Second generation women are born in The Netherlands with at least one of the parents born in Curaçao. Immigrant women of all origines have a higher TOP rate (23) compared to native Dutch women (5.5). The TOP rate of Antillean women in the Netherlands is the highest of all immigrant women (41.0). The Antilleans living in the Netherlands mainly originate from Curaçao. Comparing TOP rates of women in Curaçao with Curaçao women in the Netherlands might reveal the influence of socio-cultural factors on TOP rates (Wijsen, 2008; Stuart, Van der Wal, & Schilthuis, 2002; Lamur, 1993). The Dutch socio-cultural influence with high levels of sex education and open access to contraceptives does expect lower TOP rates in the second generation of Antillean women in the Netherlands.

The aim of this study has been to obtain strong information about the number of TOP performed by GPs in Curaçao, about characteristics of the women involved and about complications after TOP. At last the TOP rates of Curaçao are compared with those of Antillean women living in the Netherlands.

## 2. Methods

### 2.1 Registration of TOP

In the small island community, the 11colleagues performing TOP on request are well known among the GPs. All 11 were approached by the principal investigator and were invited to take part in a prospective registration study in the period from 1 November 2008 to 1 November 2009. All gave their informed consent to participate in this study. Nine of them agreed to use the registration questionnaire of the National Abortion Registration (Landelijke Abortus Registratie) from the Netherlands. Two GPs registered only the numbers of performed TOP. Although one GP indicated to be afraid of retaliatory measures of the government, none of the GPs were afraid of prosecution. The non-legal situation did not influence the willingness to cooperate and to register. In order to collect also data on the number of women having a TOP not performed by one of these 11 GPs, all 102 GPs and the eight gynaecologists received a three-monthly email in which they were requested to report any knowledge of TOP not performed by these 11 GPs. Only five TOPs were registered in this way. TOPs carried out by gynaecologists for fetal or maternal reasons were not included in this study. Approval for the study had been obtained from the Medical Ethical Committee of Curaçao. The data of TOP of Antillean women living in the Netherlands came from the Dutch National Abortion Registration.

### 2.2 Definitions

The TOP rate is calculated by dividing the number of registered TOP by the number of women between 15-45 years, multiplied by 1,000. The TOP ratio is the number of registered TOP per 100 (known) pregnancies.

### 2.3 Questionnaire

The National Abortion Registration (Landelijke Abortus Registratie) is a validated questionnaire in The Netherlands (TOP Registration form Netherlands.doc). It has been used since 1973 by the Ministry of Health Care. The questionnaire got some minor adaptation to the situation in Curaçao. Adaptations have been made on some socio demographic characteristics, referrals, type of follow up and complications and treatments after TOP (TOP registration form Curacao.doc).

### 2.3 Number of Complications in Case of TOP

The gynaecologists registered cases of complications from all TOP on request, performed by GPs as well as those of self-induced TOP during the year of study. Moreover, during three months the Emergency Department registered visits of women with complaints or complications after TOP. A checklist was used to prevent double registrations. Women with complications were included in the total number of TOP.

## 3. Results

### 3.1 Registration of TOP

1126 TOP was registered: 666 with completed questionnaires and 460 without questionnaires, only counts (including the five cases not performed in the normal circuit). With 30,000 women aged between 15 and 45 years the estimated TOP rate was 38 (TOP rate = (1126/30.000) x 1000= 38) (Population and Housing Census 2001). In 2009 the number of births was 2000, so the TOP ratio (the number of TOP per 100 (known) pregnancies) was 36. (TOP ratio = 1126/(1126 +2000) x100 = 36).

What are the characteristics of the group of 666 women with a complete questionnaire? (see Table 1) Remarkable is the short pregnancy duration on the moment of TOP. Half of the women were less than seven weeks pregnant; a third of the women did not have children. Half of the women did not have a TOP before; almost a third had one and a fifth had two or more TOPs before. The mean age of the women was 26.9 years; the age group 20 through 24 years was the most frequently represented; 35% of the women were above 30 years; teenage girls (age 14-19) represented one-fifth of the number of TOPs. The women were relative well educated. Almost half of the women who requested TOP had more than ten years of schooling; less than 10% had no more than elementary education. Two third had their own income. The majority was not married. Two third of the women originated from Curaçao. There were more medical than instrumental TOPs. Previously the instrumental outnumbered the medical method. The most frequent cause of unintended pregnancy was not using reliable contraception. When reliable modern contraceptives were used, half of the women reported inconsistent use.

**Table 1:** Results of registration of TOP on request, November 2008- 2009

		**number known results**			**number known results**
**duration of pregnancy**		**619**	**civil status**		**578**
< 7 wk	53.6%	332	married	12.1%	70
7-12 wk	41.0%	254	married, lives alone	2.2%	13
> 12 wk (-13 wk)	5.4%	33	unmarried, with parents/fam	40.5%	234
			unmarried, alone or w. children	18.3%	106
**number of children**		**666**	unmarried, lives w partner	23.9%	138
0	33.0%	220	divorced/widow	3.0%	17
1	25.8%	172			
2	24.3%	162	**nationality**		**596**
3	11.0%	73	Curacao	63.9%	381
>3	5.9%	39	Bonaire/Aruba	4.7%	28
			Venezuela	1.2%	7
**number of previousTOP**		**666**	Colombia	4.7%	28
0	53.1%	353	Dominican Rep.	8.6%	51
1	28.8%	191	Haiti	2.2%	13
2	11.0%	73	Other Caribbean area	6.7%	40
>2	7.2%	48	Surinam	1.2%	7
			Netherlands	4.0%	24
**age**	**mean age**	**656**	Rest world	2.8%	17
	**26.9 yrs**				
< 19	19.7%	129	**method**		**626**
20-24	23.9%	157	medical	59.3%	371
25-29	21.2%	139	instrumental	40.7%	255
30-34	16.0%	105			
35-39	13.3%	87	**previous method**		**313**
>40	5.9%	39	medical	29.7%	93
			instrumental	70.3%	220
**number of years education**		**602**			
<7	7.6%	46			
between 7 and 10	35.7%	215	**contraception**		**666**
between 11 and 14	33.2%	200	no contraception	63.5%	423
>14	9.8%	59			
not completed	13.6%	82	**contraception failed reason:**	36.5%	243
**source of income**		**554**	incorrect use	16.1%	39
social benefits	1.6%	9	method failed	20.2%	49
own income	64.1%	335	inconsistent use	50.1%	122
spouse/partner	10.5%	58	other	3.3%	8
parents	20.6%	114	unknown	10.3%	25
other	3.3%	18			

### 3.2 Complications of TOP

In the year of research fourteen women were admitted in the hospital with complications of TOP. 11 of the 1126 women performed by a GP (1%): six after instrumental and five after medical TOP. There were three more women with complications after self induced TOP with misoprostol (table 2). The most important complications were pain and/or prolonged blood loss, sometimes with significant fall in hemoglobine level; in two of the 14 cases, blood transfusions were required. Curettage was performed on all these women, except in one case in which there was a wait and see policy after a medical TOP. In the case of one woman the abortion debris was infected. Two ectopic pregnancies were detected after provision of TOP by a GP: one after an instrumental TOP, the other after a medical TOP. Besides the fourteen mentioned cases, three women had complications without need for hospital admission. They were seen at the Emergency Department and referred to the general practitioner for further assistance. There were no double registrations of complications of the Emergency Department and the Department of Gynaecology.

**Table 2:** Hospital admissions due to complications of TOP on request, November 2008– November 2009

Number	Age	PG	Pregnancy duration (days)	Method	Complication	Action
1	39	G4P2	?	misoprostol- self	incomplete abortion; prolonged bleeding	curettage
2	17	G1P0	97	misoprostol- self	incomplete abortion; pain	curettage
3	41	G5P3	98	misoprostol- self	incomplete abortion; prolonged bleeding	curettage
4	18	G2P1	68	methotrexate/misoprostol	incomplete abortion; hemorrhage	curettage; bloodtransfusion
5	18	G1P0	70	methotrexate/misoprostol	incomplete abortion; pain	curettage
6	19	G2P1	51	methotrexate/misoprostol	incomplete abortion	curettage
7	19	G3P2	41	methotrexate/misoprostol	abdominal pain	observation
8	26	G3P2	57	mifepristone/misoprostol	incomplete abortion	curettage
9	24	G1P0	64	instrumental	incomplete abortion; sepsis	curettage; i.v. antibiotics
10	38	G4P3	78	instrumental	incomplete abortion; hemorrhage	curettage; bloodtransfusion
11	26	G5P4	99	instrumental	incomplete abortion; pain; prolonged bleeding	curettage
12	24	G1P0	?	instrumental	incomplete abortion	curettage
13	37	G3P2	65	instrumental	incomplete abortion	curettage
14	38	G3P1	60	instrumental	incomplete abortion	curettage

## 4. Discussion

### 4.1 Insight in Background Aspects of TOP

This is the first time a prospective registration study on the prevalence of TOP in Curaçao has been carried out. The main results will be discussed and compared to national figures of TOP of Antillean women in the Netherlands.

#### 4.1.1 Gestational Age

More than half of the pregnancies were terminated before the seventh week. This means a high awareness of women to detect their pregnancy. Moreover, these women find quickly their way to a GP although TOP is not legal. Antillean women who went to the Netherlands take this awareness with them. Also over there 55 % of the Antillean women had the TOP before eight weeks amenorrhea (Wijsen, 2008).

#### 4.1.2 History of TOP per Woman

Almost 30% of the women had a history of one TOP, 11% had two previous TOPs and 7% had three or more TOPs. In the Netherlands, Antillean women have high percentages previous TOP, as has been described previously (Wijsen, 2008; Stuart, Van der Wal, & Schilthuis, 2002; Lamur, 1993). However, the total percentage of Antillean women with previous TOP in the Netherlands (53.8) is even higher than in this study (47) (Wijsen, 2008).

#### 4.1.3 Age

More than 35% of women with an unintended pregnancy are older than 30 years, compared to less than 25% of the Antillean women in the Netherlands having a TOP. This reflects probably the lack of the possibility for permanent contraception in Curaçao, as experienced in the practice of the first author. The general employee health insurance company in Curaçao (Sociale Verzekeringsbank SVB) does not include tubal ligation in its insurance package.

#### 4.1.4 Socio-Economic Status (SES)

Unmarried women in the researched group (RG) are most likely to risk a TOP. Women with more than 10 years education were over represented in our research group (RG = 43%; Pop =2 5%) as well as women with their own income (RG= 65%; Pop= 57%) (Population and Housing Census, 2001). This might be suggesting that financially autonomous women and/or higher educated women were more likely to obtain a TOP. Studies in the US show that unmarried women are more likely to obtain a TOP and women with some college education are somewhat more likely to obtain a TOP (Henshaw & Kost, 2009; Jones, Lawrence, Finer, Singh, 2010; Landry, Darroch, Singh, & Higgins, 2003). Poverty is also a risk factor for TOP according to other studies (Jones, Darroch, & Henshaw, 2002). In our research group we did not study the level of income as an SES factor. We wonder if poverty would be such a risk factor for TOP in Curaçao, because higher educated and own income women make use of TOP possibilities. It is possible that the unintended pregnant girls and women in Curaçao of lower SES are more easily in getting the child then in terminating their pregnancy. The data display more TOPs among middle and higher educated girls and women than among the lowest educated unintended pregnant girls and women. Probably the lower educated group has less incentives to plan a family size than the middle and higher educated women and girls, who are often still on high school or on an institute for higher education.

#### 4.1.5 Methods for TOP

According to the registration figures, the medical TOP method was the preferred choice (60%) in 2009. If women had a TOP previously, we notice a shift toward more medical TOP from less than 30% to nearly 60%. This reflects probably the international developments in easier access to medical TOP.

### 4.2 Antecedents for Unintended Pregnancy

Analysing the data from the 666 cases of TOP (registered questionnaires) display as main antecedent for an unintended pregnancy not using or inconsistent use of modern reliable contraceptive methods. It has been well-documented in the literature that ineffective use of contraception is related to poor sex education and to little or incorrect knowledge of sexuality and contraception (Jones, Darroch, & Henshaw, 2002; Franklin & Corcoran, 2000). School education in Curaçao is on a good level and illiteracy is relatively low, nevertheless sex education is poor. Socio-cultural influences like religious morals, lack of openness on sensitive subjects and moral standards, no debate culture, poor media information and small island social control make it difficult to provide proper sex education programs in the curriculum of (primary and secondary) schools. It is more difficult to change these things than to take high TOP rates for granted. Another antecedent contributing to the risk of unintended pregnancy are the traditional cultural notions about masculinity and gender inequality. This leads to men who still feel less responsible for the consequences of their sexual behavior and see primarily the woman and her family as responsible for care taking and raising of the child (Kocken, van Dorst, & Schaalma, 2006; Connell, McKevitt, & Low, 2004). The often young age of the unintended pregnant girl makes that her parents take over responsibility. The potential father gets also no chance to play his role or build a relationship with the coming mother of the coming baby. Another antecedent for the high rates of unintended pregnancies in Curaçao is - although reliable contraceptive methods are easily available – that there is no reimbursment for contraceptives by the health insurance companies, forming a threshold for young people and women with low incomes. This underlying study provides the perfect opportunity for policy makers and program planners to use the study’s strong data and outcomes about their own community, to revise existing practices of insufficient or lack of sex education and availability of reliable contraception to prevent unintended pregnancies.

### 4.3 Estimated Number of Total TOP according to the Number of Complications

The number of complications after TOP performed by GPs requiring hospital admission was 11 (1%). There were three cases of complications after self induced TOP among an unknown total number of self induced TOP. TOP with misoprostol is considered as relatively safe. If we extrapolate the 1% complication rate of the total researched group, we can hypothesize that the total number of self-induced TOP was not more than 300 (3 cases is 1% of the self induces TOP group) (Prasad, Kumar, & Divya, 2009; Velazco et al., 2000). The registered 1126 TOP performed by GPs together with the estimated 300 self induced TOPs (makes in total 1426) increase the TOP rate to approximately 47 and the TOP ratio to 41. With an estimated 1426 terminations of pregnancy (1126 registered, the remaining self-induced estimated by 300), the hospital admission was 10 per 1000. This is higher than in the United States where TOP is legal and accessible (3 per 1000) and similar to estimated numbers of hospital admissions in Latin America where TOP is illegal as well (Singh, 2006; Sedgh, Henshaw, Singh, Ahman, & Shah, 2007). Regarding the number of complications, under-registration should be taken into account. In a country where TOP is illegal, women presenting at a GP, at a gynaecologist or at the Emergency Department do not easy admit that they used medication to terminate a pregnancy. For example the lack of accessible reproductive healthcare was the main reason for not detecting the two ectopic pregnancies cases in our study in time, which caused extra health risk for women.

### 4.4 TOP Rate and TOP Ratio

The calculated TOP rate in Curaçao (38) is comparable with the TOP rate in the Caribbean region (35) and with the TOP rate for Antillean women in the Netherlands (41) (Alan Guttmacher Institute, 2003; Wijsen, 2008). The estimated TOP rate might be somewhat higher if estimated self induced TOP due to illegal selling of misoprostol is added (47).

The calculated TOP ratio of 36 means that, of one hundred pregnancies, 36% ended in TOP. The ratio of estimated TOP (including estimated self induced TOP) of women in Curaçao (41) is about the same as for Antillean women in the Netherlands where TOP is legal and accessible (43) (Wijsen, 2008). It seems that the illegal but institutional tolerated TOP practice in Curaçao is as easily accessible and available as the legalized TOP practice in the Netherlands. More remarkable is that 80% of the TOP among Antillean women in the Netherlands is obtained by *first* generation women and less than 20% by the *second* generation of this group of Antillean women in the Netherlands (Wijsen, 2008). This suggests that the first generation of Antillean women still decides and makes choices based on the culture and ideas from their country of origin in making use of a TOP. Opposite, the second generation of Antillean women acts different in this respect, which suggests a striking influence of sex education and accessible contraception in the country of residence, the Netherlands, and as a consequence in this respect a sharp decline of the TOP ratio (Pheterson & Azize, 2005; Bartz & Goldberg, 2009; Alan Guttmacher Institute, 2003).

### 4.5 Strengths and Limits of the Study

This study has several strengths and some limitations. One strength is the contribution to the research on the prevalence of TOP in the Caribbean, where there is not yet much tradition in this type of research on this subject. Another strength is that the data set allows a well-balanced evaluation of TOP and its complications in the Caribbean. A third strength is that the similarity between the results of Curaçao Abortion Registration in this study and the long term National Abortion Registration in the Netherlands. Besides that it could mirror the quality of the Curaçao registration instrument, it shows two cultural relationships. On the one hand the results shows *the durability* of the cultural believes from the country of origin at unintended pregnant first generation Antillean women in the Netherlands and their associated behaviors with regard to their inadequate use of contraceptives and frequent TOP, like at home. On the other hand the research outcomes give strong indications that in the long run (second generation) open *sex education* and free access to contraceptives bears fruits in terms of a strong decline of unexpected pregnancies and therefore a sharp decrease of TOP.

Limitations are partly due to the dependence of the voluntary cooperation of the GPs in the research. Of the 1126 counted TOPs 666 were extensively registered cases from nine of the eleven GPs. 455 cases came from the other two GPs and were only counted and not extensively registered. (Five cases were performed outside the normal circuit). This is a rather extensive group of the total. However there was no demographic bias between the two groups, the two GPs of the 455 cases were geographical situated between the other practices and moreover, most unintended pregnant women do not themselves choose a TOP performing GP. They are directed by their own GP or by a friend. Therefore the ‘choice’ is merely a coincidence. A further limitation might be the estimation of TOPs, self induced by illegal purchased misoprostol. Estimation via the percentages of hospitalized complication cases is not a strong source, although it is probably also not a bad indicator. A last limitation might be the lack of a more qualitative component in the research: the influence of poor sex education on the inconsistent use of reliable contraceptive methods was not qualitatively studied. Although scholarly quite some evidence is displayed on this relationship, identifying specific factors for Curacao for inadequate use of reliable contraception is important to intervene effectively and adequately (Jones, Darroch, & Henshaw, 2002).

## 5. Conclusion

This study shows that the number of TOP in Curaçao is high. The figures are similar to the numbers among Antillean women, particularly the first generation in The Netherlands (National Abortion Registration), which means that cultural related attitudes towards non-use or inconsistent use of contraception are prevalent in both groups. The second generation of Curaçao women in the Netherlands use far less TOP probably because of extensive sex education programs on school and accessible contraception in the Netherlands. It proves at the same time that improvement of contraceptive education and accessibility in Curaçao could work – like among Curaçaoans in the Netherlands, in order to bring down the high numbers of TOP. Women with completed families, who would like sterilization, were held back or were antagonized since sterilization in Curaçao is not yet reimbursed by the employees health insurance organization. Noteworthy is that the number of complications after termination of pregnancy is higher in countries where TOP is still illegal than in countries where TOP is legal. Curaçao’s TOP numbers are estimated to be similar to almost all Latin America countries, where TOP is likewise prohibited as in Curaçao. Additional research about reasons in Curaçao for inadequate use of modern contraception and interventions should be considered. For adequately monitoring future reproductive health outcomes it would be useful to regular repeat underlying prevalence research and stimulate a normal regular registration in Curaçao of TOP and its complications.

**List of abbreviations**
TOP: Termination of Pregnancy
GP: General Practitioner
RG: Research Group
